# Inactivation of the novel avian influenza A (H7N9) virus under physical conditions or chemical agents treatment

**DOI:** 10.1186/1743-422X-10-289

**Published:** 2013-09-15

**Authors:** Shumei Zou, Junfeng Guo, Rongbao Gao, Libo Dong, Jianfang Zhou, Ye Zhang, Jie Dong, Hong Bo, Kun Qin, Yuelong Shu

**Affiliations:** 1 Chinese National Influenza Center, National Institute for Viral Disease Control and Prevention, 155 Changbai Road, Beijing 102206, P.R China; 2 Key Laboratory for Medical Virology, National Health and Family Planning Commission, 155 Changbai Road, Beijing 102206, P.R China; 3 National Institute for Viral Disease Control and Prevention, China CDC, Key Laboratory for Medical Virology, National Health and Family Planning Commission, 155 Changbai Road, Beijing 102206, P.R China

**Keywords:** H7N9 virus, Inactivation, Temperatures, Ultraviolet light, pH, Disinfectants

## Abstract

**Background:**

In the spring of 2013, a novel avian-origin influenza A (H7N9) virus in Eastern China emerged causing human infections. Concerns that a new influenza pandemic could occur were raised. The potential effect of chemical agents and physical conditions on inactivation of the novel avian influenza H7N9 virus had not been assessed.

**Methods:**

To determine the inactivation effectiveness of the novel avian influenza A (H7N9) virus under various physical conditions and chemical treatments, two H7N9 viruses A/Anhui/1/2013 and A/Shanghai/1/2013 were treated by varied temperatures, ultraviolet light, varied pHs and different disinfectants. The viruses with10^7.7^ EID_50_ were exposed to physical conditions (temperature, ultraviolet light and pH) or treated with commercial chemical agents (Sodium Hypochlorite, Virkon®-S, and Ethanol) respectively. After these treatments, the viruses were inoculated in SPF embryonated chicken eggs, the allantoic fluid was collected after 72–96 hours culture at 35°C and tested by haemagglutination assay.

**Results:**

Both of the tested viruses could tolerate conditions under 56°C for 15 minutes or 60°C for 5 minutes, but their infectivity was completely lost under 56°C for 30 minutes, 65°C for 10 minutes, 70°C, 75°C and 100°C for 1 minute. It was also observed that the H7N9 viruses lost their infectivity totally after exposure of ultraviolet light irradiation for 30 minutes or longer time. Additionally, the viruses were completely inactivated at pH less than 2 for 0.5 hour or pH 3 for 24 hours, however, viruses remained infectious under pH treatment of 4–12 for 24 hours. The viruses were totally disinfected when treated with Sodium Hypochlorite, Virkon®-S and Ethanol at recommended concentrations after only 5 minutes.

**Conclusions:**

The novel avian influenza A (H7N9) virus can be inactivated under some physical conditions or with chemical treatments, but they present high tolerance to moderately acidic or higher alkali conditions. The results provided the essential information for public health intervention of novel H7N9 avian influenza outbreak.

## Background

On March 31, 2013, a novel reassortant avian influenza A (H7N9) virus (**AIV A (H7N9)**) with genomic features of low pathogenic virus causing severe infections in humans was identified in China [1]. As of Aug 20, 2013, a total of 134 human cases with avian influenza H7N9 virus infection were reported from 10 provinces and 2 municipalities in mainland China, and among them 45 died [2]. In order to minimise the animal and human health impacts, it is of crucial importance that avian influenza virus (AIV) infections in poultry are controlled. Understanding the potential effect of chemical and physical treatments on inactivation of the novel avian influenza A (H7N9) virus is significant to the implementation of proper public intervention measures. This study was designed to evaluate the effectiveness of various physical conditions (temperature, ultraviolet light (UV) and pHs), and chemical agents (Sodium Hypochlorite, Virkon®-S, and Ethanol) against avian influenza H7N9 viruses. The results of this study would provide evidences for proper virus inactivation methods and development of public hygiene measures.

## Results

### Inactivation efficacy under different heat treatments

To evaluate the effectiveness of heat treatments in inactivating the novel avian influenza H7N9 virus, 50 μl virus stock solutions containing 10^7.7^ egg infectious dose (10^7.7^ EID_50_/50 μl) viruses were treated at 56°C, 65°C, 70°C, 75°C and 100°C. As shown in Table 1, one out of six eggs of A/Anhui/1/2013(H7N9) (AH1) and three out of six of A/Shanghai/1/2013(H7N9) (SH1) showed haemagglutination (HA) positive under the treatment of 56°C for 15 min, both viruses from all six eggs survived under the treatment of 60°C for 5 min, indicating the viruses could tolerate these conditions. While the H7N9 viruses were completely inactivated to undetectable levels after 56°C for 30 min, 65°C for 10 min, 70°C, 75°C and 100°C for 1 min.

**Table 1:** Inactivation efficacy under heat treatments of the novel avian influenza A (H7N9) virus

**Temperature**	**Treatment time (minutes)**															
	**1**		**2**		**5**		**10**		**15**		**30**		**45**		**60**	
	**AH1**	**SH1**	**AH1**	**SH1**	**AH1**	**SH1**	**AH1**	**SH1**	**AH1**	**SH1**	**AH1**	**SH1**	**AH1**	**SH1**	**AH1**	**SH1**
56°C	NT*	NT	NT	NT	6/6#	6/6	6/6	5/6	1/6	3/6	0/6	0/6	0/6	0/6	0/6	0/6
65°C	NT	NT	NT	NT	6/6	6/6	0/6	0/6	0/6	0/6	0/6	0/6	0/6	0/6	0/6	0/6
70°C	0/6	0/6	0/6	0/6	0/6	0/6	0/6	0/6	0/6	0/6	0/6	0/6	0/6	0/6	0/6	0/6
75°C	0/6	0/6	0/6	0/6	0/6	0/6	0/6	0/6	0/6	0/6	0/6	0/6	0/6	0/6	0/6	0/6
100°C	0/6	0/6	0/6	0/6	NT	NT	NT	NT	NT	NT	NT	NT	NT	NT	NT	NT

**Note:** *NT=No test. #Infectivity to embryonated chicken eggs = (infected embryos)/(total embryos inoculated; n = 6) per treatment.

### Inactivation efficacy under UV irradiation exposure

To determine the efficacy of UV irradiation treatment in inactivating avian influenza H7N9 virus contaminated surface, 10^7.7^ EID_50_ viruses were spread on a Petri dish and then treated with UV irradiation in a Biosafety Cabinet for varied times at room temperature. The results showed that the viruses were inactivated after exposed to UV irradiation for 30 min or longer (Table 2). It should be noted that under UV exposure for 20 min, four out of six eggs were HA-positive with AH1 and one out of six were HA-positive with SH1. Moreover, both viruses were completely tolerant under exposure to UV for 10 min.

**Table 2:** Inactivation efficacy under UV irradiation treatment of the novel avian influenza A (H7N9) virus

**UV**	**Exposure time (minutes)**									
	**10**		**20**		**30**		**45**		**60**	
	**AH1**	**SH1**	**AH1**	**SH1**	**AH1**	**SH1**	**AH1**	**SH1**	**AH1**	**SH1**
	6/6*	6/6	4/6	1/6	0/6	0/6	0/6	0/6	0/6	0/6

**Note:** *Infectivity to embryonated chicken eggs = (infected embryos)/(total embryos inoculated; n = 6) per treatment.

### Inactivation efficacy of different pH conditions

The inactivation efficacy of the avian influenza H7N9 viruses under different pH conditions with different time intervals was shown in Table 3. The two H7N9 viruses were inactivated from an initial dose of 10^7.7^ EID_50_ to undetectable levels when exposed to pH 1–2 for 30 min or pH 3 overnight. However, the viruses still retained their infectivity when treated under pH 4–12 for 24 h or pH 3 for 30 min or 1 h. The results suggested that the novel avian influenza H7N9 virus has a strong tolerance to moderately acidic to higher alkali conditions.

**Table 3:** Inactivation efficacy of pH conditions of the novel avian influenza A (H7N9) virus

**pH values**	**Exposure time (hours)**					
	**0.5**		**1**		**24**	
	**AH1**	**SH1**	**AH1**	**SH1**	**AH1**	**SH1**
1	0/6*	0/6	0/6	0/6	0/6	0/6
2	0/6	0/6	0/6	0/6	0/6	0/6
3	6/6	6/6	6/6	6/6	0/6	0/6
4	6/6	6/6	6/6	6/6	6/6	6/6
6	6/6	6/6	6/6	6/6	6/6	6/6
8	6/6	6/6	6/6	6/6	6/6	6/6
10	6/6	6/6	6/6	6/6	6/6	6/6
11	6/6	6/6	6/6	6/6	6/6	6/6
12	6/6	6/6	6/6	6/6	6/6	6/6

**Note:** *Infectivity to embryonated chicken eggs = (infected embryos)/(total embryos inoculated; n = 6) per treatment.

### The efficacy of different disinfectants

To investigate the resistance of the novel H7N9 viruses against various disinfectants, 50 μl virus stock solutions containing 10^7.7^ EID_50_ viruses were treated with 0.5 and 1% Sodium Hypochlorite, 0.5 and 1% Virkon®-S, and 75% ethanol at room temperature respectively. As shown in Table 4, these two viruses were inactivated from an initial dose of 10^7.7^ EID_50_ to undetectable levels after only 5 min when exposed to recommended concentrations of 0.5% or 1% sodium hypochlorite, 0.5% or 1% Virkon-S and 75% ethanol.

**Table 4:** The disinfection efficacy of disinfectants of the novel avian influenza A (H7N9) virus

**Disinfectants**	**Component**	**Exposure time (minutes)**					
		**5**		**10**		**30**	
		**AH1**	**SH1**	**AH1**	**SH1**	**AH1**	**SH1**
Ethanol	75% Ethanol	0/6*	0/6	0/6	0/6	0/6	0/6
Chlorine compound	0.5% Sodium Hypochlorite	0/6	0/6	0/6	0/6	0/6	0/6
	1.0% Sodium Hypochlorite	0/6	0/6	0/6	0/6	0/6	0/6
Virkon®-S	0.5% Dipotassium Peroxodisulphate	0/6	0/6	0/6	0/6	0/6	0/6
	1.0% Dipotassium Peroxodisulphate	0/6	0/6	0/6	0/6	0/6	0/6

**Note:** *Infectivity on embryonated chicken eggs = (infected embryos)/(total embryos inoculated; n = 6) per treatment.

## Discussion

The aim of this study was to identify commonly available inactivation methods or disinfection agents that might be used for public protection against the novel avian influenza A (H7N9) virus or the decontamination methods at laboratory or field conditions. The results indicated that the novel avian influenza H7N9 viruses can be effectively inactivated by temperature treatment under 56°C for 30 min, 65°C for 10 min, 70°C, 75°C and 100°C for 1 min. But, it should be noted that the H7N9 viruses still could survive after treatment under 56°C for 15 min or 65°C for 5 min. Similar results were demonstrated with highly pathogenic avian influenza (HPAI) H5N1 virus, which was inactivated after exposure at 56°C for 30 min [3]. However, a significant amount of information is available on the resistance at high and low temperatures of AIVs but not all data are in agreement. Such as the H5N1 viruses were completely inactivated at 70°C for 60 min or at 75°C for 45 min [4]. Low pathogenic avian influenza (LPAI) H7N2 viruses were completely inactivated at 56°C after 60 min or at 60°C after 10 min, while retained their infectivity in a water bath at 56°C after 30 min [5,6]. Additional study on AIV H7N3 indicated that the virus remained infectious after being treated at 56°C condition for 30 min, but its infectivity was lost after for 60 min [7]. Our study suggested that the novel avian influenza A (H7N9) virus has not presented more tolerance to high temperature treatments.

AIV inactivation through UV light irradiation has a potential sterilizing application in the laboratories. The present study showed that UV light irradiation on the novel H7N9 virus is effective under the UV light exposure for 30 min or longer within 75 cm distance. However, the virus can survive if exposed to UV for shorter than 20 min. The result suggested that enough UV exposure time is necessary for avoiding cross-contamination on lab-works. In addition, other disinfection measures should be implemented after lab works since only microbes on the surface of material and in the air can be killed by UV light [8]. UV irradiation could be considered an appropriate method only when the surfaces are well cleaned and the source of light is positioned very close to the surfaces to be disinfected [9].

Orthomyxoviridae viruses are considered to be sensitive to acid pH values, although their retention of infectivity is dependent on degree of acidity and virus strain [10]. Our study indicated that the novel avian influenza H7N9 viruses may present a strong tolerance to both moderately acidic and higher alkali conditions. The H7N9 viruses lost their infectivity when exposed to less pH 2 for 30 min or pH 3 overnight conditions, while infectivity remained under pH 4–12 conditions for all contact times. However, previous studies suggested that different subtypes of influenza could present different tolerance to acidic or alkali conditions. LPAI H7N2 virus lost 100% infectivity under pH 2 condition for 5 min, but no effect exposure to pH 5, 7, 10 or 12 for 15 min [6]. LPAI H7H3 was unable to maintain their infectivity after exposure to pH 1, 3, 10 and 14 for 48 h [7]. The study on Thailand AIV H5N1 suggested that all ranges of pH 3, 5, 7, 9 and 12 could not inactivate the tested viruses after exposure for 5 and 10 min respectively [4].

In general, on the basis of their resistance to chemical agents, viruses can be divided into three categories (A, B and C) according to the presence/absence of lipids on the virus particle and size of virus. AIVs belong to category A, which can be inactivated easily by all major classes of disinfectants if used properly [11]. Commercially available disinfectant products evaluated in this study, including Sodium Hypochlorite, Virkon®-S and ethanol, effectively disinfect the novel avian influenza H7N9 viruses at the recommended concentrations for 5 min at room temperature. Study demonstrated that the viral RNA of two LPAI strains H5N9 and H7N3 was completely damaged after exposure to Sodium Hypochlorite at recommended concentration [12]. AIV H5N1 was completely inactivated after treatment with acid hypochloride for 10 min [13]. The efficacy of Virkon®-S against AIVs has been evaluated on H5N9 and H7N3, the results showed that the fresh Virkon®-S solution at the recommended dilution was able to destroy the viral genome making it undetectable by real time RT-PCR [12]. 0.5% Virkon®-S was able to fully inactivate AIV after 90 min while 1% or 2% Virkon®-S achieved virucidal activity after 30 min [7]. Ethanol is efficacious against AIVs and other enveloped viruses, our results demonstrated that the novel avian influenza H7N9 viruses were completely inactivated after 75% ethanol treatment for only 5 min. As AIV H7N2, which lost its infectivity after treatment with 70% ethanol for 5 min or 15 min [5,6]. However, the disinfection efficacy also depends upon the strain of the virus, exposure time, quantity of the virus and nature of the medium used.

## Conclusion

The results indicated that the novel avian influenza H7N9 viruses can be completely inactivated using high temperature (e.g. 56°C or above), UV light irradiation, and commercial disinfectants (Sodium Hypochlorite, Virkon®-S and Ethanol). But the virus presents a high tolerance to moderately acidic or higher alkali conditions. The present results would provide essential information for public health intervention of novel avian influenza H7N9 outbreaks.

## Materials and methods

### The viruses and virus propagation

In March 2013, three urban residents of Shanghai or Anhui, China, were identified to be infected with AIVs A (H7N9) [1]. Understanding the potential effect of chemical and physical treatments on inactivation of the H7N9 virus is significant to the implementation of proper public intervention measures. Two avian influenza H7N9 viruses (A/Anhui/1/2013 and A/Shanghai/1/2013) were selected to assess its tolerance to varied physical conditions or chemical reagents in this study. The viruses were propagated in 9-day-old SPF embryonated chicken eggs in a 35°C humidified incubator, and the allantoic fluid(AF) was harvested after 48 h inoculation. The virus titres, determined as EID_50_/ml, were evaluated according to the Reed and Muench method (Reed and Muench, 1938) [14]. The virus was divided into single-use tube, refrozen, and stored at −80°C until further use.

### Treatment of AIVs A (H7N9) with physical conditions and chemical reagents

#### Temperature inactivation

The avian influenza H7N9 viruses were subjected to the conditions of various temperatures. The efficacy of heat treatments inactivating H7N9 viruses was determined. The tested protocol was modified from Swayne and Beck [15]. For test of thermal inactivation, 50 μl virus stock solutions containing 10^7.7^ EID_50_ were added into thin-walled 0.2 ml polypropylene thermocycler tubes (Axygen scientific, USA) and placed in a precision tube-holding heating block of the thermocycler with heated lid (Eppendorf Mastercycler, Germany) at different temperatures, including 56, 65, 70 and 75°C for 5, 10, 15, 30, 45 and 60 min and additionally, 70°C, 75°C and 100°C for 1 and 2 min. The treated viruses were placed in an ice bath immediately to stop the heat treatment and 450 μl PBS was added for inoculation of SPF embryonated chicken eggs.

#### UV inactivation

50 μl virus stock solution containing 10^7.7^ EID_50_ was spread on Petri dish (Corning Incorporated, 35 mm × 10 mm) surface using 10 μl tip (Axygen scientific, USA) and exposed to UV light (distance of 75 cm and wavelength of 250-270 nm) in Biosafety Cabinet (Sterilgard® III advance, the Baker Company) at room temperature. The cover of the Petri dish was removed when samples were exposed to the UV light, 450 μl PBS was added to the Petri dish after UV exposure for 10, 20, 30, 45 and 60 min respectively. And then the recovery of the virus was tested immediately by inoculation of SPF embryonated chicken eggs.

#### The tolerance of AIVs A (H7N9) on varied pH conditions

To determine the tolerance of the novel virus on the acidic and alkali conditions, the viruses AH1 and SH1 were exposed to different pH values (1–12) for different time intervals at room temperature, as described in previous study with some modifications [16]. 50 μl virus stock solutions containing 10^7.7^ EID_50_ were mixed thoroughly with 450 μl PBS with pH 1, 2, 3, 4, 6, 8, 10, 11 and 12, adjusted by using sodium hydroxide or hydrochloric acid, and treated for 0.5, 1 and 24 h at room temperature respectively. And then the recovery of the virus was tested immediately by inoculation of SPF embryonated chicken eggs.

#### Disinfectants inactivation

Commercial disinfectants, including Sodium Hypochlorite, Virkon®-S (Antec™ International, UK) and ethanol, were tested in this study. The tested protocol was modified from Suarez et al. [12]. Briefly, all disinfectants were diluted with distilled water following the manufacturers’ recommendation. 50 μl virus stock solutions containing 10^7.7^ EID_50_ were mixed thoroughly with 450 μl of each working disinfectant, 4.5 ml PBS was added to the mixture to stop the disinfection after 5, 10, and 30 min treatment at room temperature respectively. And then the recovery of the virus was tested immediately by inoculation of SPF embryonated chicken eggs.

#### Virus inactivation testing in SPF embryonated chicken eggs

All treated viruses were inoculated into 9-day-old SPF embryonated chicken eggs to determine their survival. Each of the virus suspension exposed to physical conditions (temperature, ultraviolet light and pH) was made 10-fold dilution with PBS, 100-fold dilution for the untreated virus stock solution as positive control, 10-fold dilution for each disinfectant control. Each diluted samples was filtered through 0.22 μm filter (Milliplex™, Millipore corp., Bedford, MA, USA) before inoculation into six 9-day-old SPF embryonated chicken eggs for 72-96 h. Embryo mortality observed within 24 h post inoculation was considered nonspecific and discarded. Allantoic fluid (AF) was collected from each egg and checked for HA as the sign of viral growth in the eggs.

#### Biosafety levels

In view of the biosafety issues involved in handling avian influenza H7N9 viruses, all the experiments were conducted in a biosafety level 3 facility for this study.

## Abbreviations

AIV A (H7N9): A novel reassortant avian-origin influenza H7N9 virus; AIV: Avian influenza virus; UV: Ultraviolet light; EID50: 50% egg infectious dose; AH1: A/Anhui/1/2013(H7N9); SH1: A/Shanghai/1/2013(H7N9); HA: Haemagglutination assay; HPAI: Highly pathogenic avian influenza; LPAI: Low pathogenic avian influenza; SPF: Specific pathogen-free; AF: Allantoic fluid.

## Competing interests

The authors declare no competing interests. The contents of this article are solely the responsibility of the authors and do not necessarily represent the views of China CDC and other organizations.

## Authors’ contributions

YLS designed the research and draft the paper; SMZ and JFG performed the research; SMZ and RBG joined into the designation of the study, analysed the data and draft the paper; LBD,YZ, JD and HB helped with titration of the virus; JFZ and KQ helped to draft the manuscript. All authors read and approved the final manuscript.
